# A Review of Herbal Therapy in Multiple Sclerosis

**DOI:** 10.15171/apb.2018.066

**Published:** 2018-11-29

**Authors:** Sina Mojaverrostami, Maryam Nazm Bojnordi, Maryam Ghasemi-Kasman, Mohammad Ali Ebrahimzadeh, Hatef Ghasemi Hamidabadi

**Affiliations:** ^1^Young Researchers and Elite Club, Behshahr Branch, Islamic Azad University, Behshahr, Iran.; ^2^Department of Anatomy & Cell Biology, Faculty of Medicine, Mazandaran University of Medical Sciences, Sari, Iran.; ^3^Cellular and Molecular Research Center, Department of Anatomy & Cell Biology, Faculty of Medicine, Mazandaran University of Medical Sciences, Sari, Iran.; ^4^Cellular and Molecular Biology Research Center, Health Research Institute, Babol University of Medical Sciences, Babol, Iran.; ^5^Pharmaceutical Sciences Research Center, School of Pharmacy, Mazandaran University of Medical Sciences, Sari, Iran.; ^6^Immunogenetic Research Center, Department of Anatomy & Cell Biology, Faculty of Medicine, Mazandaran University of Medical Sciences, Sari, Iran.

**Keywords:** Multiple sclerosis, Inflammation, Demyelination, Remyelination, Herbal therapy

## Abstract

Multiple sclerosis is a complex autoimmune disorder which characterized by demyelination and axonal loss in the central nervous system (CNS). Several evidences indicate that some new drugs and stem cell therapy have opened a new horizon for multiple sclerosis treatment, but current therapies are partially effective or not safe in the long term. Recently, herbal therapies represent a promising therapeutic approach for multiple sclerosis disease. Here, we consider the potential benefits of some herbal compounds on different aspects of multiple sclerosis disease. The medicinal plants and their derivatives; Ginkgo biloba, Zingiber officinale, Curcuma longa, Hypericum perforatum, Valeriana officinalis, Vaccinium macrocarpon, Nigella sativa,Piper methysticum, Crocus sativus, Panax ginseng, Boswellia papyrifera, Vitis vinifera, Gastrodia elata, Camellia sinensis, Oenothera biennis, MS14 and Cannabis sativa have been informed to have several therapeutic effects in MS patients.

## Introduction

Multiple sclerosis (MS) is an autoimmune disease that mostly occurs in young adulthood.^1^ The etiology of MS disease is still not well understood, but both genetic and environmental factors were found to have important roles in MS disease initiation or progression.^2^ In MS disease, inflammatory cells demolish myelin sheath in the CNS which weakens action potential conduction.^3^ Two cardinal properties of MS are acute inflammation that associated with demyelination and another one is axonal loss.^4^ After injury, oligodendrocyte precursor cells (OPCs) which are residing at parenchyma continuously produce myelinating oligodendrocytes.^3-5^ In addition, regarding to the ability of neural stem cells for differentiation to OPCs, these stem cells are considered as an important source for remyelination.^6-8^ These endogenous stem cells proliferate, migrate and differentiate to OPCs after brain injuries. However, endogenous OPCs can produce myelin and improve some aspects of the MS disease, but endogenous repair may fail in long term.^7,9^ Therefore, several studies have focused on different approaches (including targeting specific signaling pathways, stem cell therapy, suppressing the inflammation process and reprogramming of glial cells to OPCs …) that improve myelination.^10^ Despite the potential benefits of stem cell therapy in the improvement of myelin repair,^11^ its clinical application has been hampered because of the possibility of teratoma formation, cell rejection and ethical problems.^12,13^ Therefore, there is still a need for developing new drugs which have no or less considerable side effects.

The pathophysiology of MS is not well elucidated, which makes itsʼ treatment strategy very difficult and perplexing.^14^ At the present time, most of the strategies in MS treatment are focused on preventing of inflammation in the CNS.^15^ Interferon beta (IFN-beta) was firstly confirmed as an effective drug for treatment of MS in 1993.^16^ Afterward, different drugs were introduced for curing MS such as glatiramer acetate, natalizumab, alemtuzumab and fingolimod.^14^ All of these mentioned drugs were partially effective and their remarkable adverse effects makes them unsuitable for proloned use.^17^ For example, several studies indicated the adverse effects of IFN-beta consumption including, stroke, headache, migraine and depression.^17^ Until now, no absolute treatment has been found for MS, therefore, trying to find a completely effective and safe treatment is still ongoing.

Use of complementary and alternative medicine (CAM), in particular herbal remedies have noticeably risen in MS patients over the last decades.^18,19^ Herbal therapy is known as a helpful strategy for curing the different disorders from ancient to the present time.^20^ Previous studies reported that medicinal plants have several therapeutic effects in different disorders such as cancers, diabetes and neurodegenerative diseases.^21,22^ Recently, a growing number of findings have indicated that some herbal compounds improve myelin repair and lead to suppression of inflammation.^23,24^ Also, there are many studies that reported the anti-inflammatory and antioxidant effects of medicinal plants, as well as other helpful properties which make them as a natural, safe and reliable remedy for treatment of neurodegenerative diseases.^25,26^ Perivious studies have indicated that MS patients are interested in using herbal medicines to control their disease symptoms.^26^ For example in China, Chinese herbal medicine (CHM) is widely used by MS patients for ameloriating the severity of disease.^27^ The beneficial effects of CHM in MS disease is occured by reduction of the severity of MS disease; including antioxidative properties, anti-apoptotic effects, anti-inflammatory properties and promoting the differentiation of local stem cells to myelin producing cells.^28^ MS patients usually use CAM, and medicinal plants as a member of this family plays a crucial role to cure MS and itsʼ associated symptoms.^29^ Different herbal medicines are recommended for MS patients, but the understanding of their efficacy is not well described. In this review; we will discuss some of herbal compounds beneficial effects on MS disease (Table 1 and Table 2). We conducted a search for all English language articles in Google Scholar, Science Direct, Scopus, PubMed and Medline for medicinal plants, that have been used for their therapeutic potentials in MS disease, studies which their publication dates from January 1960 to April 2018 were used.

**Table 1:** Summary of herbal medicines used in the treatment of Multiple sclerosis

**Plant**	**Author (Country)**	**Design of study**	**Dosage**	**Number**	**Duration of study**	**Effects**	**Ref**
***Ginkgo *** ***biloba***	Johnson et al (USA)	Clinical trial-Double-blind, placeo-controlled	240 mg/day of ginkgo extract	22 MS patients	4 weeks	Treatment with ginkgo extract relieved fatigue with no adverse effect in MS patients	34
	Lovera et al (USA)	Clinical trial-Double-blind, placeo-controlled	240 mg/day of ginkgo extract	38 MS patients	12 weeks	Improvement of the cognitive performance were reported in treated group	37
	Brochet et al (France)	Clinical trial-Double-blind, placeo-controlled	240 and 360 mg/day of ginkgolide B	104 MS patients	1 week	ginkgolide B was not an effective treatment for exacerbations of MS	180
***Zingiber*** ***officinale***	Jafarzadeh et al (Iran)	EAE model of MS in mice	200 and 300 mg/kg ginger extract	24	4 weeks	Ginger extract ameliorated EAE severity and modulated the expression of IL-27, IL-33	43
***Curcuma longa***	Xie et al (Japan)	EAE model of MS in rats	100 and200 mg/kg curcumin extract	21	2 weeks	Curcumin decreased the inflammation and the severity of EAE	53
	Natarajan and Bright (USA)	EAE model of MS in SJL/J mice	50 and 100 µg curcumin in 25 µl DMSO / day	-	4 weeks	Curcumin decreased CNS inflammation and demyelination also,decreased the severity of EAE	54
	Mohajeri et al (Iran)	EAE model of MS in rats	12.5 mg/kg of curcumin	20	17 days	Treatment with polymerized nano-curcumin decreased the severity of EAE and increased the remyelination	23
***Oenothera*** ***biennis***	Firouzi et al (Iran)	Double blind, randomized clinical trial	18–21 g/day Evening primrose oil and *C. sativa* oils	100 MS patients	24 weeks	Treatment with co-supplemented *C. sativa* and *evening primrose* oils decreased the clinical score in MS patients	158
	Horrobin (Canada)	Double blind, randomized clinical trial	-	14 MS patients	24 weeks	Treatment with colchicine and evening primrose oil improved manual dexterity test and clinical score in MS patients	159
***Hypericum*** ***perforatum***	Naziroglu et al (Turkey)	In-vitro study on neutrophils of MS patients	20 μM/ml *H. perforatum* for 2 hours	9 MS patients	-	Treatment with *H. perforatum* indicated the protective effects on oxidative stress in MS patients	69
***Vaccinium*** ***macrocarpon***	Gallien et al (France)	Double blind, clinical trial, placebo-controlled	36 mg/day Cranberry extract(proanthocyanidins)	171 MS patients	1 year	Treatment with cranberry extract versus placebo did not prevent UTI occurrence in MS patients	81
***Nigella sativa***	Fahmy et al (Egypt)	EAE model of MS in rats	2.8 g/kg *Nigella sativa extract*	22	4 weeks	*N. sativa* ameliorated the clinical signs of EAE, suppressed inflammation and enhanced remyelination in the CNS	85
	Noor et al (Egypt)	EAE model of MS in rats	2.8 g/kg *Nigella sativa extract*	22	4 weeks	*N. sativa* suppressed inflammation in EAE rats. Also, *N sativa* enhanced remyelination in the cerebellum and reduced the expression of TGF *β*1	87
***Crocus *** ***sativus***	Ghaffari et al (Iran)	EAE model of MS in rats	Intrahippocampal (5and 10 μg/rat) injection of the saffron	35	3 days	Local injection of saffron extract modulated the oxidative stress markers (reduced the activity of GPx and SOD enzymes), through scavenging of ROS	103
	Ghazavi et al (Iran)	EAE model of MS in C57bl/6 mice	100 µL saffron extract	20	3 weeks	Treatment with saffron decreased inflammation in the spinal cord and decreased the severity of EAE	102
***Panax*** *** ginseng***	Hwang et al (Korea)	EAE model of MS in C57bl/6 mice	200 μg of an acidic polysaccharide of *Panax ginseng*	-	33 days	Acidic polysaccharide of *Panax ginseng* decreased the infiltration of inflammatory cells in the CNS, also suppressed EAE score by inhibiting the proliferation of T cells and the production of inflammatory cytokines	113
	Etemadifar et al (Iran)	Randomized Double-blind, placeo-controlled	250 mg ginseng tablets	52 MS patients	12 weeks	Ginseng treatment had no adverse effect on MS patients as well as reduced fatigue and had a positive effect on quality of life	114
***Boswellia*** ***papyrifera***	Sedighi et al (Iran)	Randomized, double-blinded, placebo-controlled study	600 mg of *B. papyrifera*	80 MS patients	8 weeks	*B. papyrifera* improved the visuospatial memory of MS patients	124
***Vitis*** ***vinifera***	Sato et al (USA)	EAE model of MS in C57bl/6 mice	20 mg/kg per day	-	8 weeks	Resveratrol treatment worsened the demyelination and inflammation without neuroprotective effects in the CNS	129
	Kelly et al (USA)	EAE model of MS in C57bl/6 mice	100 and 250 mg/kg Sigma resveratrol	-	4 weeks	Resveratrol delayed the onset of EAE and had a significant neuroprotective effect as well as prevents neuronal loss	130
	Shindler et al (USA)	EAE model of MS in SJL/J mice	500 and 1000 mg/kg resveratrol	62	4 weeks	Resveratrol treatment prevented neuronal loss during optic neuritis and reduced neurological dysfunction during EAE	131
***Camellia *** ***sinensis***	Mahler et al (Germany)	Randomized, double-blinded, placebo-controlled study	600 mg/d EGCG	18 MS patients	12 weeks	Treatment with EGCG improved muscle metabolism during moderate exercise in MS patients	155
**MS14**	Tafreshi et al (Iran)	EAE model of MS in C57bl/6 mice	MS14 containing 30% of the diet	14	20 days	*Treatment with MS14 *ameliorated the clinical signs of EAE and reduced neuropathological changes	163
	Kalan et al (Iran)	EAE model of MS in C57bl/6 mice	MS14 containing 30% of the diet	25	35 days	MS14 decreased EAE symptoms and lymphocyte infiltration into the CNS	164
	Kalan et al (Iran)	EAE model of MS in C57bl/6 mice	MS14 containing 30% of the diet	25	35 days	Treatment with MS14 reduced clinical signs of EAE, demyelination and IL-6 production	165
***Cannabis sativa***	Zajicek et al (UK)	Randomized, placebo-controlled trial	Capsules containing 2.5 mg of THC and 1.25 mg of cannabidiol	630 MS patients	15 weeks	cannabinoids improved patients' mobility and improved in spasticity	173
	Zajicek et al (UK)	Double blind, placebo controlled, phase III study	Capsules containing cannabidiol 0.8–1.8 mg and 2.5 mg THC	279 MS patients	12 weeks	Treatment with cannabinoids improved the relief from muscle stiffness in MS patients	172
	Wade et al (UK)	Double-blind, randomized, placebo-controlled study	120 mg cannabidiol and 120 mg THC	160 MS patients	10 weeks	Treatment with cannabinoids improved patient's spasticity, without any adverse effects	174
	Greenberg et al (USA)	Double-blind, randomized, placebo-controlled study	Smoking one marijuana cigarette containing 1.54% THC	10 MS patients	3 days	Smoking marijuana improved eyes-open and eyes-closed tests, and noise variance values	178
	Brady et al (USA)	Open-label, pilot study	2.5 mg of THC and 2.5 mg CBD per spray	10 MS patients	8 weeks	Cannabinoids decreased urinary urgency, number and volume of incontinence episodes, frequency and nocturia in MS patients. Also, spasticity and quality of sleep improved significantly in the treated group	179

**Table 2:** Use of herbal medicines in Multiple sclerosis, according to the symptomatic problems

**Usage**	**Plant**	**References**
**Antidepressant**	*Hypericum perforatum* *Crocus sativus*	69,101
**Sleeping problem**	*Piper methysticum* *Valeriana officinalis*	73,93
**Improvement in cognitive impairment**	*Ginkgo biloba* *Boswellia papyrifera*	36,124
**Urinary system dysfunction**	*Vaccinium macrocarpon* *Cannabis sativa*	79,179
**Fatigue**	*Ginkgo biloba* *Panax ginseng*	114,34
**Anti-inflammatory and neuroprotective**	*Ginkgo biloba* *Zingiber officinale* *Curcuma longa* *Oenothera biennis* *Nigella sativa* *Crocus sativus* *Panax ginseng* *Boswellia papyrifera* *Vitis vinifera* *Gastrodia elata* *Camellia sinensis* *Cannabis sativa* MS14	33,34,87,101,113,124,130,140,134,159,164,167

### Ginkgo biloba

*Ginkgo biloba* L. (well-known as ginkgo), is one of the oldest living tree species from the family *Ginkgoaceae.* Ginkgo is native to Korea and China, but now it can be found all over the world.^30^ Ginkgo refers to an extract of the leaves of the *G. biloba* trees, which traditionally used as a remedy to improve the mental alertness and memory.^31^ The current reputation of *G. biloba *can be attributed to a pioneeering research that informed *G. biloba* is an effective cure for cognitive issues.^32^

Studies have shown that anti-inflammatory and inhibiting the platelet-activating factor (PAF) properties of ginkgo extract (EGB761) are effective on MS disease.^33,34^ Ginkgolides is the major component of *G. biloba*, its effect on the PAF activity represents the possible therapeutic role of this plant on MS.^34^ PAF’s role in inflammation process is obviously introduced, so ginkgo can inhibit this process.^35^ In addition, ginkgo reverses cognitive impairment and reduces fatigue in MS patients.^36,37^*G. biloba* is generally safe with no side or adverse effects^38^ but in some few cases, dizziness, headaches and ocular bleeding associated with its usage.^30^ Consumption of* G. biloba* is almost safe and has obvious therapeutic effects for MS patients; improving functional status (fatigue) of people with MS and neuroprotective activities are the valuable medical properties of *G. biloba*.

### Zingiber officinale

*Zingiber officinale* Roscoe (Ginger), is an aromatic plant in the family Zingiberaceae. *Z. officinale *is native to India and commonly grown in Asia, tropical Africa and Latin America.^38^ Ginger root is routinely used as an aromatic spice and a traditional drug.^39^ Recent studies acknowledged the anti-cancer,^40^ antioxidant^41^ and anti-inflammatory activities of ginger.^42^

Anti-inflammatory capacity of ginger is the reason of its consumption by MS patients.^43^ Gingerols and its dehydrated derivatives (shogaols), are the major components of the ginger that exhibit anti-inflammatory effects.^44^ The anti-inflammatory effects of 6-shogaol were reported by inhibiting the expression of inducible nitric oxide synthase (iNOS) and cyclooxygenase- 2 (COX-2) in macrophages, as well as preventing dopamine reduction and reducing apoptose rate in CNS.^45^ 10-gingerol is another important component of ginger which attribituted with anti-inflammatory effect in fresh ginger.^46^ 10-gingerols inhibits LPS-induced NO and production of pro-inflammatory cytokines by inhibiting the NF-jB activation.^46^ Positive effects of ginger and itsʼ active compounds (6-shogaol and 10-gingerol) were approved in animal models of MS, by exerting anti-inflammatory and neuroprotective effects, but still clinical studies on MS patients are needed to confirm these results.

### Curcuma longa

*Curcuma longa* L. is a tropical plant, native to southern and southeastern tropical Asia.^47^ *C. longa* is belonging to the ginger family, *Zingiberaceae*. A yellow coloring-matter obtained from the roots of* C. longa*, is named curcumin.^47^ Curcumin is widely used as a dietary spice and pigment. Curcumin is commonly consumed as an Asian folk remedy for treating biliary disorders, anorexia, cough, sinusitis and sore throat.^48^ Studies indicated that curcumin exerts a wide range of biological activities, including anti-inflammatory,^49^ antitumor^50^ and antioxidants effects.^51^

Anti-inflammatory effect of curcumin has been obviously examined in different studies.^44,52^ Curcumin does its anti-inflammatory role in two major ways: (1) inhibition of pro-inflammatory cytokines and (2) inhibition of Th17 differentiation and its related pathways.^52^ Curcumin ameloriated the severity of experimental autoimmune encephalomyelitis (EAE), as a animal model of MS disease, also reduced the infiltration of inflammatory cells to the CNS.^52^ Curcumin modulates inflammatory process by decreasing the expression of the different pro-inflammatory and inflammatory cytokines.^44,53^ In one study, polymerized form of nano-curcumin was used for treating EAE model of MS, which demonstrated anti-inflammatory and antioxidative effects as well as increasing remyelination and decreasing EAE score.^23^ Furthermore, curcumin can decrease the secerity of MS disease by blocking IL-12 signaling pathway in T cells.^54^ Side effects of curcumin is associated with high dose consumption which causes nausea and diarrhea.^55^ Although, therapeutic effects of curcumin were demonstrated in different studies but human studies are certainly needed to confirm the recommendation of curcumin in MS patients.

### Hypericum perforatum

*Hypericum perforatum* L*.* better known as St John’s Wort is a flowering plant from the family *Hypericaceae. H. perforatum* is a native plant in Europe and Asia but today has worldwide spread.^30^ From the ancient time, *H. perforatum* was popular plant because it had a wide range of therapeutic effects for different diseases such as anxiety, depression and menstrual disorders.^56^ Currently, *H. perforatum* is used for treating the inflammation-related disorders,^57^ cancers^58^ and neurodegenerative diseases.^59^

The usage of *H. perforatum* in MS disease is related to its antidepressant effects.^60,61^ Hypericin is the main antidepressant component of *H. perforatum* which stimulates capillary blood flow in brain.^62^ Studies have showed that Hypericin strongly inhibits the MAO enzymes and has a strong affinity for Sigma receptors which regulate dopamine level.^63^ Recent studies have shown that hypericin acts as an antagonist for adenosine, benzodiazepine and GABA receptors.^64^ Many studies emphasis on another constituent, Hyperforin, for the therapeutic effects of this plant. It has been reported that Hyperforin has antidepressant effects as a result of inhibiting the uptake of dopamine, serotonin, noradrenaline, GABA, and L-glutamate.^65,66^ There are some clinical studies which have shown the effectiveness of *H. perforatum* on depression conditions.^60,67,68^* H. perforatum* can be recommended to MS patients due to its antidepressant, antioxidative and anti-inflammatory effects.^69^

### Valeriana officinalis

*Valeriana officinalis* L. (Valerian) is native to Europe, North America and parts of Asia.^68^ Valerian is a plant in the family of *Caprifoliaceae,* the root and rhizome of this herb are used for different medicinal purposes.^68^ In ancient Greek, valerian had various medicinal applications, for example, for digestive problems, epilepsy and urinary tract infections.^70^ In addition, valerian was introduced as a treatment for sleeping problems and insomnia.^71^

Sleep disturbance is the most important cause of fatigue in MS patients.^72^ Clinical trials demonstrated that major component of *V. officinalis* root extract, valerenic acid, is effective in treatment of mild-to-moderate sleeping disorders.^73^ Like benzodiazepines which are GABA-analogs, valerenic acid has particular affinity for the GABA_A_ receptor.^30^ Limited adverse effects such as stomachache and allergic reaction have been observed in patients under treatment of valerenic acids.^30,74^ Consumption of *V. officinalis* can be suggested to MS patients due to the ameliorating effects on sleeping problems and fatigue status.

### Vaccinium macrocarpon

*Vaccinium macrocarpon* (Cranberry, Large Cranberry), is a North American species of cranberry in *Ericaceae* family*.* Cranberry juice is obtained from the *V. macrocarpon* fruit.^75^ Cranberry has been traditionally used for treatment of bladder and kidney disorders by Native Americans.^75,76^

MS patients are susceptible to the urinary tract colonizations (UTC) and urinary tract infections (UTIs), as a result of the bladder dysfunction in MS disease.^77^ Studies have shown that cranberry juice or its produced-capsules is a beneficial remedy for treatment of UTIs.^78^ It has been reported that cranberry can inhibit *Escherischia coli* adherence to the urethra.^79^ Cranberry has two substantial compounds, fructose and proanthocyanidin, that stick to the fimbriae of *E. coli* and effectively hindering the bacteriaʼs ability to attach to the urethra.^79^ There are a few clinical studies that examined the preventive effects of cranberry on UTIs in MS patients.^80^ However, in different studies cranberry was found to be effective against urinary tract infections, but in one clinical trial of MS patients, it not showed acceptable prevention from urinary tract infections.^81^

### Nigella sativa

*Nigella sativa* L. usually known as black seed is widely used as a medicinal plant belongs to the family *Ranunculaceae.* Black seed is native to Southern Europe, Southwest Asia and North Africa.^82^ Seeds and oil of *N. sativa* has a long historical and religious background for treatment of various illnesses such as headache, back pain and gastrointestinal problems.^83,84^ Several studies have shown that black seed has diverse therapeutic effects, including anticancer, analgesic, antimicrobial, anti-inflammatory, renal protective and antioxidant properties.^84,85^ Thymoquinone is the active compound of *N. sativa* seeds.^86^

There are several evidences that indicated the anti-inflammatory capacity of black seed oil.^85,87^ In vitro studies demonstrated the inhibitory effects of *N. sativa* oil and its active compound, thymoquinone, on the production of inflammatory mediators‏ {such as IL-1b, IL-6, TNF-a, IFN-c and PGE2}.^88,89^ In addition, Black seed oil inhibited COX and 5-LO pathways of arachidonate metabolism.^89,90^ Thymoquinone also potently inhibiting the non-enzymatic peroxidation in brain phospholipid liposomes.^84^ The therapeutic effects of *N. sativa* in animal models of MS were reported. *N. sativa* enhanced remyelination in CNS, reduced inflammation processes and suppressed the expression of TGF *β*1 in EAE models of MS disease.^87^

### Piper methysticum

Piper methysticum (kava kava) is a psychoactive herb in the family *Piperaceace*, which has been used in the Pacific Islands for hundreds of years. The root of this plant is well-known for its sedative and anesthetic properties.^80^ Kava traditionally used for producing a comforting and relaxing drink.^80,91^

Combination of kava and valerian seems to be more effective for treatment of stress-induced insomnia than either herb alone.^92^ The active compound of kava is cavalactones, which interacts with GABA_A_ receptors and decreases anxiety and sleeping problems.^93,94^ Kava also can potentiate the sedating effects of the drugs that usually used in MS disease such as Lioresal (Baclofen).^95^ Extensive use of kava may lead to hepatotoxicity, hepatic necrosis and cholestasis hepatitis.^96^ It can be concluded that kava alone or in combination with other herbal medicines is a promising candidate for treating anxiety disorders of MS patients.

### Crocus sativus

*Crocus sativus* L. (Saffron) is a flowering plant in the family *Iridaceae*. Saffron stigma has been widely used as a medicinal plant for healing the different disorders.^97^ Saffron has been commonly used as an herbal drug for its sedative, stimulant and anticatarrhal properities.^97,98^ Several studies suggested that saffron can be effective in treatment of hypertension and memory impairments. Additionally, saffron has been demonstrated anti-inflammatory and antitussive effects.^99^ Crocetin and crocin are the two main active compounds of saffron stigma, which have a wide range of therapeutic activitis.^100^

Antidepressant and anti-neuroinflammatory effects of saffron are evidently effective in MS disease.^101-103^ Crocin exerts its anti-inflammatory effects via inhibiting syncytin-1 and nitric oxide (NO)-induced astrocyte and oligodendrocyte cytotoxicity, also decreases neurological injuries in experimental autoimmune encephalomyelitis (EAE).^104,105^ Syncytin-1 is highly expresses in microglia, astrocytes and glial cell of MS lesions.^106^ Studies have shown that crocin has antidepressant effects in mild to moderate depression.^99^ Excessive consumption of saffron induces dizziness, nausea, vomiting and diarrhea.^107^ Depression is a common condition in MS disease which adversely affect health status. According to this point, antidepressant activity of saffron can be highly helpful in depressive disorders of MS patients.

### Panax ginseng

*Panax ginseng* also known as Asian ginseng is a traditional herbal medicine in Asia for thousands of years, which belongs to the *Araliaceae* family.^108^ Ginseng root is traditionally used in powdered form to regenerate the body and mind, increase physical strength and prevent aging.^109^ The main active compound of *P. ginseng* is ginsenosides, that exhibits anti-inflammatory, antioxidant, and anti-apoptotic properties.^110,111^In addition, *P. ginseng* is one of the most useful medicinal plants for curing different neuroinflammatory diseases such as Parkinson’s disease, Alzheimer’s disease, Huntington’s disease and Multiple sclerosis.^112^

Some evidences have indicated that ginseng can decrease the inflammation and fatigue, which may be useful in MS patients.^113,114^ Ginseng reduced the severity of EAE by inhibiting the proliferation of T cells, inhibiting the production of the inflammatory cytokines (FN-γ, IL-1β and IL-17) and depleting of CD25+ cells.^113^ In clinical studies ginseng led to fatigue improvement and had a positive effect on quality of life.^114^ Excessive intake of ginseng can lead to several adverse effects including hypertension, insomnia, rashes and diarrhea.^115^ We can deduce that ginseng is an effective remedy for curing MS-related fatigue and enhancing quality of life in these patients.

### Boswellia papyrifera

*Boswellia papyrifera* belongs to the *Burseraceae* family.^116^ Gum production through resin of* B. papyrifera*, has high economic value.^117^ The resin of *B. papyrifera* has been traditionally used in treatment of ulcers, chronic inflammation and for memory support.^118^ The main active compound of *B. papyrifera* resin is boswellic acids.^119^ Several studies have indicated the different therapeutic effects of boswellic acids such as anti-inflammatory, antitumor and antioxidant effects.^120,121^

Cognitive impairment is a common clinical symptom in MS patients, with incidence rates up to 70%.^122^ Cognitive deficits in MS patients affect various aspects including attention, information processing efficiency, processing speed, long term memory and visual learning.^123^ Anti-inflammatory and neuroprotective properties of *B. papyrifera*, reversed the cognitive impairments in MS patients.^124^ In one clinical trial, patients with MS which received *B. papyrifera*, had a significant visuospatial memory improvement compared to the control group.^124^ Administration of *B. papyrifera* enhances cognitive impairment in MS patients, but still there is a need for large scale trials to completely clarify the therapeutic effects of *B. papyrifera* in MS patients.

### Vitis vinifera

*Vitis vinifera* L. (common grape vine) is one of the most important fruit crops in the world from the family *Vitaceae. V. vinifera* is cultivated in the most countries of Europe, Northern Africa and Western Asia.^125^ The leaves and seeds of this plant have various medicinal properties. Resveratrol (*trans*-3, 4, 5-trihydroxystilbene) is a phenolic compound that produced in the grapes in response to injuries of fungal pathogens.^126^ Resveratrol has been reported to have several pharmaceutical effects such as anti-inflammatory, anticancer, antioxidant and antiviral properties.^127,128^

Both neuroprotective and anti-inflammatory effects of resveratrol were informed in several researches.^129-131^ Resveratrol exhibits neuroprotective effects by inhibiting the microglia activation and decreasing the pro-inflammatory factors production through the MAPKs, phosphoinositide3-kinase (PI3-K)/Akt, glycogen synthase kinase-3β (GSK-3β) and NADPH oxidase signaling pathway.^127,132,133^ Resveratrol can cross the blood–brain barrier (BBB), therefore it can be an ideal candidate for treating neuroinflammatory and neurodegenerative diseases.^129,134^ Excessive intake of common grape causes gastrointestinal side effects including nausea, abdominal pain, flatulence and diarrhea.^135^ Neuroprotective effects of *V. vinifera* were demonstrated in different neurodegenerative diseases, but more specific studies on MS disease are needed to determine itʼs therapeutic role in MS.

### Gastrodia elata

*Gastrodia elata* Blume (tianma) is a saprophytic herb from the family *Orchidaceae*. *G. elata* is a traditional Chinese plant, native to the oriental countries.^136^ Tianma is the dried rhizome of the *G. elata* which were used as a traditional herbal medicine for a variety of conditions such as headaches, vertigo and hypertension.^137^ In addition, tianma is commonly used for the treatment of neurodegenerative disorders and memory improvement.^138,139^

Due to neuroprotective and anti-neuroinflammatory effects of *G. elata,* it can be considered as a promising candidate for MS therapy. *G. elata* reduces oxygen free radicals and protects against neuronal damage.^137,140,141^ *G. elata* indicated anxiolytic-like properties via the GABA-ergic nervous system.^142^ It has been reported that *G. elata* has protective effects against global ischemia, nitric oxide synthase activity and apoptosis.^143^ Gastrodin is the main active component of the tianma which mediates its neuroprotective effects.^144^ Vanillin and Benzyl alcohol are the other active compouds of *G. elata* that have anti-inflammatory effects by inhibiting the generation of reactive oxygen species (ROS) and inhibiting the activities of cyclooxygenase- (COX-) 1 and COX-2.^145^*G. elata* plays an important protective role in the neurorestorative processes, therefore it can be a helpful remedy for MS patients. however, future animal models and clinical studies of MS disease are needed to clarify the possible therapeutic effects of this plant on MS patients.

### Camellia sinensis

*Camellia sinensis* L. is well known as green tea, one of the oldest beverages in the world from the *Theaceae* family.^146,147^ Dried leaves of *C. sinensis* are used in green tea production.^147^ Green tea is used for several different purposes including weight loss, cardiovascular disorders, inflammation and neuroprotective effects.^148^

Green tea exhibits anti-inflammatory and neuroprotective properties.^149,150^ Epigallocatechin-3-gallate (EGCG) is one of the most important active compounds of green tea which is attributed to anti-inflammatory and neuroprotective properties of this plant.^134^ EGCG is a polyphenol compound that inhibits the production of inflammatory mediators such as TNF-α, IL-1β and IL-6 and improvs the neuroprotection in nervous system.^151,152^ In other studies, EGCG inhibited LPS-induced microglial activation and protected against inflammation-mediated dopaminergic neuronal injury.^153^ Regular and habitual consumption of green tea is safe, but, consuming high doses causes liver toxicity.^154^‏ Green tea has anti-inflammatory effects which can protect CNS from neurodegenerative diseases such as MS. Also, green tea regulates energy expenditure in the body which may relief MS-related fatigue.^155^

### Oenothera biennis

*Oenothera biennis L*. (Evening primrose) is a species in the family of *Onagraceae*. Evening primrose oil is produced from the seeds of *O. biennis* which has numerous pharmacological effects.^156^ The traditional consumptions of this plant was for the treatment of swelling in the body, then were used for other problems such as skin disorders, gastro-intestinal disorders and asthma.^157^ Two parts of the plant; flowers and seeds, are used for extracting the oils.

The application of Evening primrose oil in treatment of MS is related to its anti-inflammatory and immune-modulating effects.^158,159^ These beneficial effects largely related to the evening primrose oil abundant supply of polyunsaturated fatty acids (PUFA) content.^159^ Gamma-linolenic acid is a precursor of prostaglandin and it is responsible for anti-inflammatory effects of evening primrose oil.^158^ In clinical trials, Evening primrose oil led to significant performance improvement in the manual dexterity test.^159^ In another clinical study gamma-linolenic acid rich oil had an obvious effect in relapsing-remitting MS, which meaningfully decreased the relapse rate and the progression of disease.^160^ However, more clinical trials are needed to prove the therapeutic effects of Evening primrose oil in MS patients.

### MS14 (Penaeus latisculatus, Apium graveolens and Hypericum perforatum)

MS14 is an Iranian natural herbal-marine drug, which has obvious therapeutic effects on MS patients.^161,162^ MS14 consists of 90% *Penaeus latisculatus*, 5% *Apium graveolens* and 5% *Hypericum perforatum.*^163^ In both animal models and human clinical trials, MS14 was effective in treatment of MS.^162,164^ Antioxidant and anti-inflammatory effects, are the two features of MS14 that halt the progression of MS disease.^161^ MS14 showed some benefits on quality of life and improvement of patients' mobility (lower limb).^162^ The neuroprotective and anti-inflammatory properties of MS14 were reported by suppressing the proliferation responses of T cells and decreasing the expression levels of IL-6, IL-5, IL-10, TNF-α and IL-1β. Furthermore, MS14 inhibited the inflammatory cell infiltration into the CNS and up-regulated LCN2 in all stages of EAE.^164,165^ Oral consumption of MS14 was reported properly safe without any adverse effects.^162^ MS14 is an herbal-marine drug that has beneficial effects in MS disease and other neroudegenerative disorders, but still more studies are needed to find various mechanisms and pathways that MS14 shows itʼs neuroprotective effects.

### Cannabis sativa

*Cannabis sativa* L. (Bang, Marijuana, and Hachis) is a flowering plant in the genus *Cannabis* from the *Cannabaceae* family. *C. sativa* is native to Western and Central Asia, but extensively cultivated in Asia and Europe.^166^*C. sativa* has traditionally been used for healing the different diorders such as allergies, inflammation and sexually transmitted diseases.^167^ Seeds, leaves and flowers of *C. sativa* was reported to have various therapeutic and medicinal values. There are numerous studies that indicated the pharmacological usage of *C. sativa,* including analgesic effect, anticancer activity, anti-inflammatory activity, central nervous system depressant activity and immunomodulatory effect.^166^

*C. sativa* is the most important and the most common plant which is using for treating of MS. There are numerous studies that indicated cannabinoids consumption reduced muscle stiffness, bladder disturbance, spasms, neuropathic pain and sleep disorders in MS patients.^168,169^ The major compound of *C. sativa* is ∆-9tetrahydrocannabinol (THC).^170^ THC binds to cannabinoid receptors (CBR) in the CNS and acts as a partial agonist to CB1 and CB2 receptors.^171^ THC was shown to have anti-inflammatory and neuroprotective properties.^167^ Some studies have shown the applications of cannabinoids for improvments of spasticity in MS, consumption of cannabinoids led to significant improvement in patient-reported spasticity.^172-174^ Also, the combination of THC and cannabidiol (CBD) was more effective for treatment of moderate to severe refractory spasticity in MS patients.^175^ Cannabinoids and their synthetic drugs indicated attractive anti-inflammatory effects in animal models of MS.^176^ Synthetic cannabinoids can reduce inflammation by suppressing the TNF-α production in the brain. Also, Synthetic cannabinoids can improve motor function by preventing the infiltration of immune cells into the CNS, and can decrease the pro-inflammatory cytokines secretion such as IFN-µ, IL-17, IL-6, IL-1β and TNF-α.^167^ Sativex is a combination of the two derived cannabinoids of the *C. sativa*; THC and CBD, which has been formulated for oromucosal mouth spray to relief neuropathic pain, spasticity, sleeping difficulties, bladder disturbance and other symptoms associated with MS.^177^ Orally administration of THC can decrease the intensity of several signs and symptoms of MS such as, decreasing spasticity, rigidity, tremor, as well as improving walking ability, performance of handwriting and bladder control.^169^ Smoking marijuana in patients with MS demonstrated to have some advantageous effects, including healing the spasticity, pain, tremor and emotional dysfunction.^178^ In one clinical trial, cannabinoids decreased the urinary symptoms, urinary incontinence, frequency of urination and nocturia in treated group.^179^ There are some adverse effects associated with *C. sativa* such as the risks of cancer, cardiovascular disease, nausea, vomiting and impaired driving.^61^

## Conclusion

Medicinal plants have opened a new horizon in curing neurodegenerative disorders such as Parkinson's disease, AD and MS. literature data review indicated that herbal medicines could be effective in the treatment of MS disease and itsʼ related symptoms, by reducing the demyelination, improving remyelination and suppressing the inflammation in the CNS. On the basis of the above mentioned review, it can be concluded that the anti-inflammatory effect is the main reason of medicinal plants therapeutic effects in MS disease, through which medicinal plants ameliorate the severity of disease and reduce neuropathological changes. Anti-inflammatory effects of medicinal plants usually occur through inhibiting the inflammatory cell infiltration into the CNS, decreasing the production of pro-inflammatory and inflammatory cytokines. Further studies are needed to disclose the exact mechanisms of action, through which medicinal plants exhibit their anti-inflammatory and neuroprotective effects. Given that most studies of herbal therapy effects in MS have been done on animal models, still there is a great need for approving these studies by clinical trials to recommend these mentioned plants for MS patients. In addition to neuroprotective effect, medicinal plants have other beneficial effects for MS patients, such as sedation, improving sleep quality, anti-depressant effects, relief muscle stiffness and reducing bladder disturbance.

## Ethical Issues

Not applicable.

## Conflicts of Interest

All authors declare no conflicts of interest.
